# The Cuban Propolis Component Nemorosone Inhibits Proliferation and Metastatic Properties of Human Colorectal Cancer Cells

**DOI:** 10.3390/ijms21051827

**Published:** 2020-03-06

**Authors:** Yahima Frión-Herrera, Daniela Gabbia, Michela Scaffidi, Letizia Zagni, Osmany Cuesta-Rubio, Sara De Martin, Maria Carrara

**Affiliations:** 1 Department of Pharmaceutical and Pharmacological Sciences, University of Padova, L.go Meneghetti 2, 35131 Padova, Italy; yahima81@gmail.com (Y.F.-H.); daniela.gabbia@unipd.it (D.G.); michela.scaffidi@studenti.unipd.it (M.S.); letizia.zagni@studenti.unipd.it (L.Z.); maria.carrara@unipd.it (M.C.); 2 Chemistry and Health Faculty, Technical University of Machala, Ave. Panamericana Vía a Pasaje Km. 5 1/2, Machala 070101, Ecuador; osmanycuesta@yahoo.com

**Keywords:** propolis, nemorosone, colorectal cancer, apoptosis cell death, epithelial–mesenchymal transition

## Abstract

The majority of deaths related to colorectal cancer (CRC) are associated with the metastatic process. Alternative therapeutic strategies, such as traditional folk remedies, deserve attention for their potential ability to attenuate the invasiveness of CRC cells. The aim of this study is to investigate the biological activity of brown Cuban propolis (CP) and its main component nemorosone (NEM) and to describe the molecular mechanism(s) by which they inhibit proliferation and metastatic potential of 2 CRC cell lines, i.e., HT-29 and LoVo. Our results show that CP and NEM significantly decreased cell viability and inhibited clonogenic capacity of CRC cells in a dose and time-dependent manner, by arresting the cell cycle in the G0/G1 phase and inducing apoptosis. Furthermore, CP and NEM downregulated *BCL2* gene expression and upregulated the expression of the proapoptotic genes *TP53* and *BAX*, with a consequent activation of caspase 3/7. They also attenuated cell migration and invasion by inhibiting MMP9 activity, increasing E-cadherin and decreasing β-catenin and vimentin expression, proteins involved in the epithelial–mesenchymal transition (EMT). In conclusion NEM, besides displaying antiproliferative activity on CRC cells, is able to decrease their metastatic potential by modulating EMT-related molecules. These finding provide new insight about the mechanism(s) of the antitumoral properties of CP, due to NEM content.

## 1. Introduction

Colorectal cancer (CRC), one of the most common oncological disease worldwide, is an aggressive cancer with metastatic behavior [1,2,3]. Approximately 40%–60% of CRC patients develop metastases in other organs, in particular liver and lungs, which represent the main sites of CRC metastasis [4]. Although surgical resection of metastasis can here be often performed, metastatic CRC remains one of the principal causes of cancer-related morbidity and mortality [5,6,7].

Tumor cells acquire the capacity to infiltrate blood or lymphatic vessels through an epithelial to mesenchymal transition (EMT) process, which increases cell migration and invasion, intravasation and extravasation. Taken together, these processes result in increased tumor aggressiveness and metastasis [8,9]. Therefore, the proteins associated to cell migration and invasion, as well as the mechanisms involved in EMT, represent a pharmacological target for the treatment of CRC patients [10,11].

The pharmacological therapy of CRC involves biological or synthetic drugs able to suppress or prevent cancer progression [3,12,13]. In this context natural products, widely studied by the scientific community, are considered an interesting option, especially when they are safe and inexpensive [13,14]. Several clinical studies have shown that plant-based products exert anticancer effects by numerous mechanisms, i.e., the induction of cell cycle arrest and cell death by apoptosis or necrosis, the activation of DNA repair systems, the regulation of signaling pathways involved in cancer progression, and the inhibition of cancer invasion and metastasis [15,16,17,18].

It is known that many bee products, such as royal jelly, honey, pollen, propolis and bee venom, display antitumor and chemo-preventive activity and can be evaluated as alternative strategies for cancer treatment [19,20,21,22]. Propolis, a natural resinous bee product, has been used since ancient times as a traditional remedy for the treatment of various diseases, and in recent years its anticancer potential has been demonstrated [23,24,25,26]. The therapeutic effects of propolis depend on its chemical composition, which is related to the geographical area of origin and the environmental conditions of its production [27,28,29]. Several studies have shown that brown Cuban propolis (CP) exerts antiproliferative and cytotoxic properties on different cancer cell lines [30,31,32]. The chemical profiling of this type of propolis indicated that nemorosone (NEM), a prenylated benzophenone, is the main component of brown CP [33,34,35] to which the biological properties of this propolis can be attributed [36,37,38]. In light of these considerations, in this study we investigate the effect of brown CP and NEM alone on the proliferation, migration and invasion of two human CRC cell lines, LoVo and HT-29. Moreover, we evaluated for the first time their effect on the regulation of some epithelial markers involved in EMT process.

## 2. Results

### 2.1. NEM and CP Suppress CRC Cell Viability

**Figure 1:**
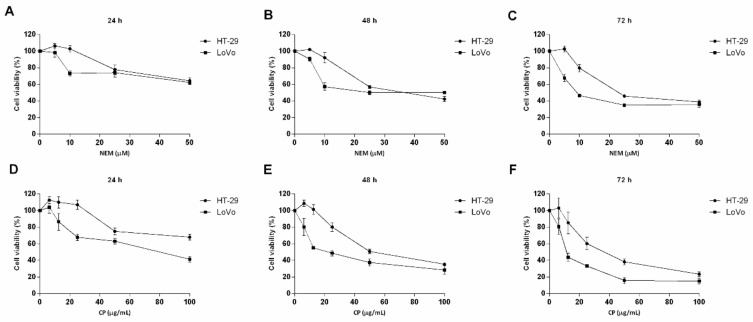
Effect of NEM and CP on CRC cell viability. HT-29 and LoVo cells were exposed to increasing concentrations of NEM (**A**–**C**) and CP (**D**–**F**) for 24, 48 and 72 h. Data are presented as mean ± S.D. of three independent experiments.

Colorimetric MTT assay was used to assess the effect of CP and NEM on the viability of HT-29 and LoVo cell lines. Dose-response curves of both CRC cell lines exhibited a dose- and time- dependent decrease in cell viability (Figure 1). Moreover, these curves indicated that HT-29 cells were more sensitive to all the treatments compared to LoVo cells. However, no significant differences were observed between the two CRC cell lines regarding the IC_50_ values (Table 1), thus indicating that HT-29 and LoVo cells have a similar susceptibility to NEM and CP effects. As already demonstrated by previous studies ([32] and refs. therein), considering NEM content in CP, the IC_50_ obtained in this study confirm that the antiproliferative effect of CP on CRC cell lines is mainly due to NEM. 

### 2.2. Inhibitory Effect of NEM and CP on Clonogenic Capacity of CRC Cell Lines

**Figure 2:**
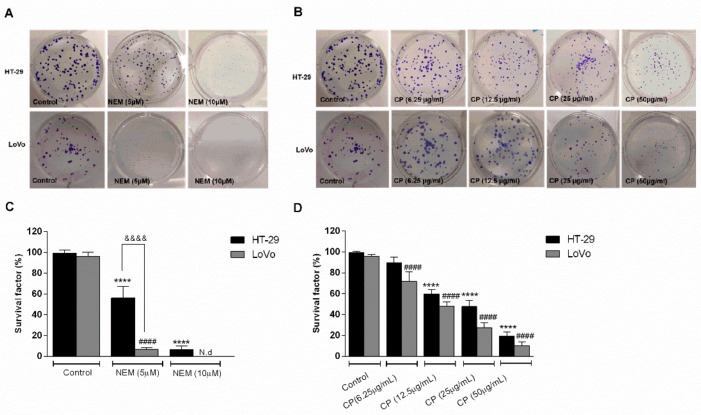
Effect of NEM (**A**,**C**) and CP (**B**,**D**) on the clonogenic capacity of colorectal cancer (CRC) cells. Magnification: 10×. Data are expressed as survival factor and represent the mean ± S.D. of three independent experiments. **** *p* < 0.0001 vs. untreated cells. LoVo cells: ^####^ *p* < 0.0001 vs. untreated cells. ^&&&&^ *p* < 0.0001 *vs* the other cell line treated with the same conditions. N.d: not detected.

The capacity of CRC cell lines to survive and create new colonies after treatment with NEM or CP for 10 days was determined by a clonogenic assay. As shown in Figure 2, the survival factors of HT-29 and LoVo cells were significantly reduced after NEM and CP treatment in a concentration-dependent manner. At the two highest NEM concentration (25 and 50 µM), no colonies were observed (data not shown). Accordingly, at CP concentrations higher than 50 µg/mL, no colonies were observed. Moreover, LoVo cells were more sensitive than HT-29 to a 10 day-exposure to NEM. These findings further confirmed that brown CP and its main component NEM reduce the survival of CRC cell lines. 

### 2.3. NEM and CP Induce G1 Phase Cell Cycle Arrest in CRC Cell Lines

In order to obtain additional information about cell growth inhibition mechanism of CP and NEM on HT-29 and LoVo cells, we performed further experiments using the IC_50_ values obtained by the dose-response curves after 72 h of treatment (reported in Table 1).

**Figure 3:**
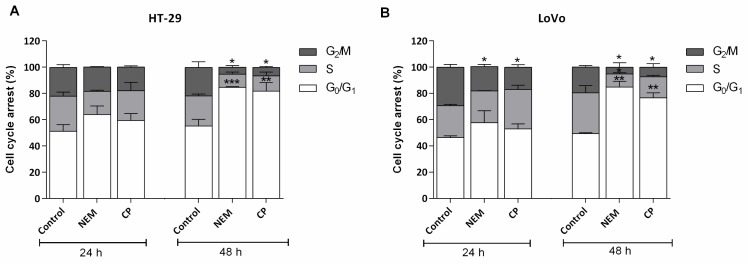
Effect of cell cycle distribution of (**A**) HT-29 and (**B**) LoVo cells exposed to NEM (IC_50_) and CP (IC_50_) for 24 and 48 h. Results are presented as mean ± S.D. of three independent experiments. * *p* < 0.05, ** *p* < 0.01, *** *p* < 0.001 vs. untreated cells.

Flow cytometry was used to investigate the effect of NEM and CP on CRC cell cycle distribution after 24 h- and 48 h-treatment. As shown in Figure 3, the percentage of HT-29 and LoVo cells in G0/G1 phase tended to increase only slightly after 24 h of treatment, while this increase became significant after 48 h of exposure. Furthermore, after 48 h, the number of HT-29 cells in G0/G1 phase increased from 51.9% of the control to 84.6% and 81.82% of NEM- and CP-treated cells, respectively. Conversely, for the LoVo cell line, this increase ranged from 49.28% of the control to 84.74% in NEM- and 76.65% in CP-treated cells, respectively. Moreover, this marked increase of cells in the G0/G1 phase was accompanied by a decrease in that of cells in the G2/M phase. These findings indicate that NEM and CP induced a significant cell cycle arrest of CRC cell lines in the G0/G1 phase after 48 h of exposure.

### 2.4. NEM and CP Induce Apoptosis in CRC Cell Lines

**Figure 4:**
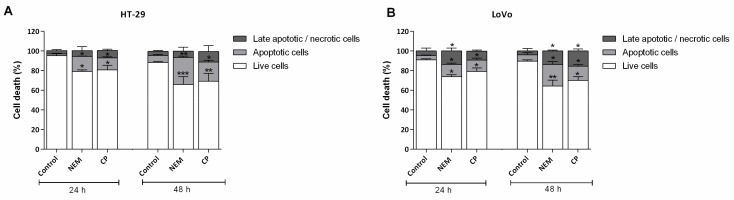
Effect of NEM (IC_50_) and CP (IC_50_) on apoptosis of HT-29 (**A**) and LoVo (**B**) cell lines. Data are presented as mean ± S.D. of three independent experiments. * *p* < 0.05, ** *p* < 0.01, *** *p* < 0.001 vs. untreated cells.

The percentage of apoptotic cell after NEM or CP treatment (IC_50_/72 h) for 24 and 48 h was determined by flow cytometry using Annexin V-FITC/PI. As shown in Figure 4, there was a significant increase in apoptotic cells in both cell lines after a 24 h- and 48 h-treatment after both treatments. The percentage of apoptotic cells significantly increased as exposure time increased in both HT-29 (24 h: Control—1.83%, NEM—15.26%, CP—12.73%, 48 h: Control—7.14%, NEM—27.71%, CP—19.47%) and LoVo (24 h: Control—4.36%, NEM—12.46%, CP—11.36%, 48 h: Control—6.38%, NEM—21.94%, CP—14.27%) cells.

### 2.5. NEM and CP Regulate the Expression of Apoptosis-related Genes in CRC Cell Lines

**Figure 5:**
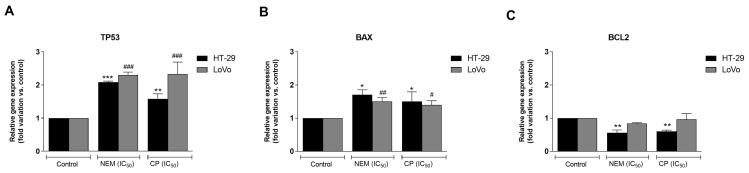
Effect of NEM and CP (IC_50_/72 h) on apoptosis-related genes of CRC cell lines. The mRNA levels of P53 (**A**), BAX (**B**) and BCL2 (**C**) are expressed as fold of change vs. control. Data are expressed as mean ± S.D. of three independent experiments. HT-29 cells: *p < 0.05, ** *p* < 0.01, *** *p* < 0.001 vs. untreated cells. LoVo cells: ^#^ *p* < 0.05, ^##^ *p* < 0.01, ^###^ *p* < 0.001 vs. untreated cells.

The expression of apoptosis-related genes was evaluated in HT-29 and LoVo cell lines after a 24 h-treatment with NEM or CP (IC_50_/72 h). CRC cells exhibited a significant increase of the mRNA levels of the pro-apoptotic genes *TP53* and *BAX* with respect to untreated cells (Figure 5A,B). Furthermore, mRNA levels of the antiapoptotic gene *BCL2* were downregulated by NEM and CP (Figure 5C) in HT-29 cells, while it did not change after treatment in LoVo cells. These results suggest that CP and NEM cause *TP53*-mediated apoptosis in both cell lines.

### 2.6. NEM and CP Promote the Activation of Caspases in CRC Cell Lines

**Figure 6:**
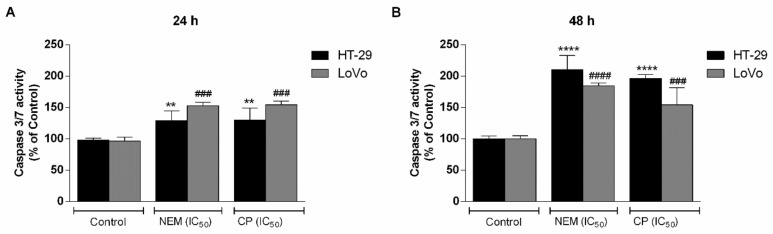
Activation of caspase 3/7 in CRC cell lines after a 24 h-(**A**) and 48 h-(**B**) treatment with NEM or CP (IC_50_/72 h). The caspase 3/7 activity is expressed as percentage of fold change with respect to untreated cells. The values are normalized to the amount of living cells. Data are expressed as mean ± S.D. of three independent experiments. HT-29 cells: ** *p* < 0.01, **** *p* < 0.0001 vs. untreated cells. LoVo cells: ^###^ *p* < 0.001, ^####^ *p* < 0.0001 vs. untreated cells.

The Caspase-Glo 3/7 assay was performed to evaluate the effect of NEM and CP on the activation of caspases 3 and 7 in HT-29 and LoVo cell lines. Both NEM and CP (IC_50_/72 h) caused a marked activation of caspase 3 and 7 in CRC cells (Figure 6). Furthermore, the activation of caspases increased significantly with exposure time in HT-29 (24 h: NEM—129.34%, CP—129.27%; 48 h: NEM—210.31%, CP—196.44%) and LoVo cells (24 h: NEM—150.38%, CP—154.33%; 48 h: NEM—186.67%, CP—154.06%). No significant differences were observed in caspase activation between the two cell lines. These findings indicate that this mechanism played a pivotal role in the apoptosis induced by NEM and CP in CRC cell lines. 

### 2.7. NEM and CP Inhibited Migration and Invasion of CRC Cell Lines

**Figure 7:**
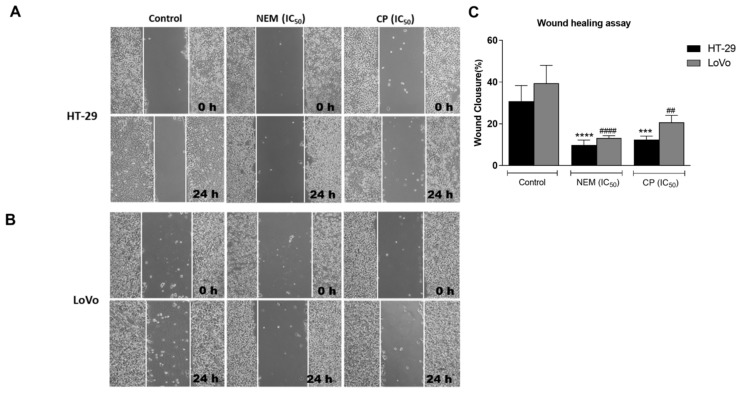
Wound healing assay in the CRC cell lines HT-29 (**A**) and LoVo (**B**; magnification 10×). Quantitative analysis of scratch wound healing assay after a 24 h-treatment with NEM or CP (IC_50_/72 h; **C**). Data are expressed as mean ± S.D. of three independent experiments. HT-29 cells: *** *p* < 0.001, **** *p* < 0.0001 vs. untreated cells. LoVo cells: ^##^ *p* < 0.01, ^####^ *p* < 0.0001 vs. control.

Since one of the peculiar features of CRC is its metastatic and invasive capacity [39], the wound healing assay and transwell migration and invasion assays were performed in HT-29 and LoVo cells treated with NEM or CP (IC_50_/72 h) for 24 h. As shown in Figure 7, wounds after 24 h of exposure to NEM and CP were wider than in untreated cells, indicating the inhibitory effect of NEM and CP on the motility of CRC cells. 

**Figure 8:**
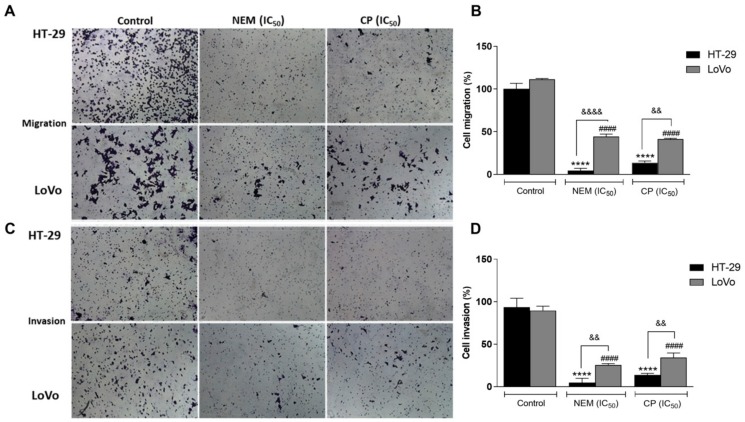
Effect of NEM and CP (IC_50_/72 h) on cell migration (**A**,**B**); magnification 10×) and invasion (**C**,**D**). Data are expressed as mean ± SD of three different experiments. HT-29 cells: **** *p* < 0.0001 vs. control. LoVo cells: ^####^ *p* < 0.0001 vs. control. ^&&^ *p* < 0.01, ^&&&&^ *p* < 0.0001 vs. the other cell line treated with the same conditions.

Furthermore, the results of the transwell assay with/without Matrigel after 24 h of treatment indicated that both NEM and CP significantly reduced the migration and invasion capability of HT-29 and LoVo cells with respect to untreated cells (Figure 8). Interestingly, the two cell lines displayed significantly different susceptibility in their migration/invasion capacity to NEM and CP treatment, probably because of the differences of their genetic background, which have been associated with their migratory and invasive properties [40,41] 

### 2.8. NEM and CP Regulate Expression of MMP9 and EMT Protein Markers in CRC Cell Lines

**Figure 9:**
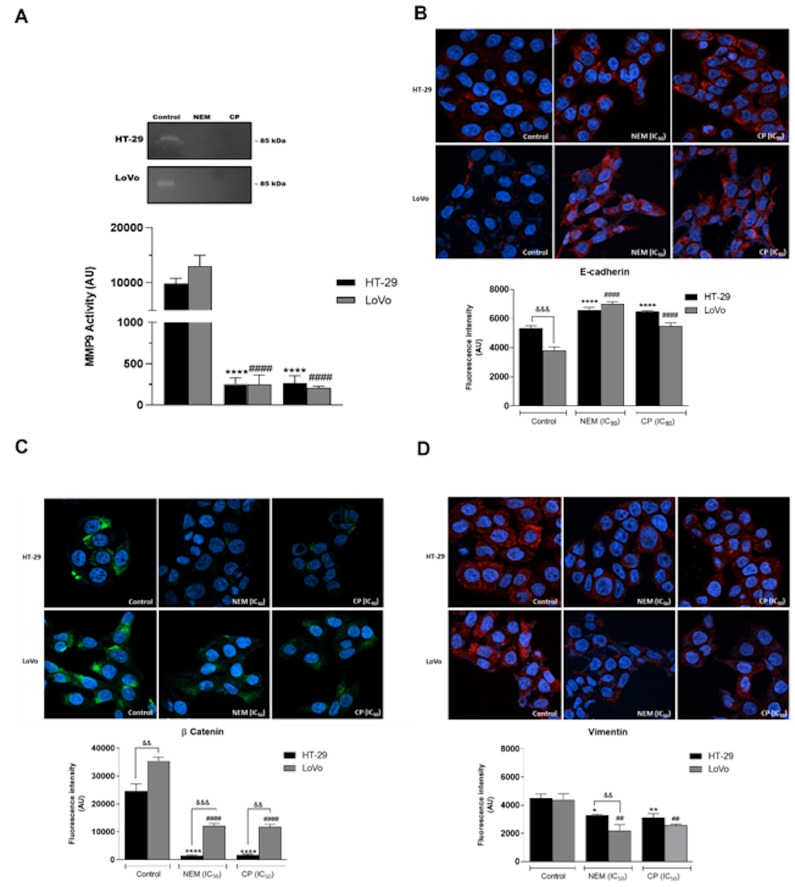
Effect of NEM and CP on proteolytic activity of MMP9 in CRC cells (**A**). Effect of NEM and CP (IC_50_/72 h) on the expression of epithelial–mesenchymal transition (EMT)-related markers E-cadherin (**B**), β-catenin (**C**) and vimentin (**D**) in CRC cells. In immunocytochemistry (ICC) images, E-cadherin and vimentin are in red and β-catenin is in green. Nuclei were stained with Hoechst (blue). Magnification: 40×. Data are reported as the mean ± SD of three independent experiments. ** *p* < 0.01, **** *p* < 0.0001 vs. untreated cells. LoVo cells: ^####^ *p* < 0.0001 vs. untreated cells. ^&&^ *p* < 0.01, ^&&&^ *p* < 0.001 vs. the other cell line treated with the same conditions. N.d: not detected.

In order to confirm the role of brown CP and its main component NEM in regulating CRC metastasis, MMP9 activity and the expression of several EMT markers, including E-cadherin, cell membrane β-catenin and vimentin, were assessed by zymography and immunofluorescence respectively, after a 24 hour-treatment. As shown in Figure 9A, there was a significant reduction in MMP-9 activity in CRC cell lines after treatment with NEM and CP (IC_50_/72 h). Moreover, both treatments significantly increased E-cadherin expression (Figure 9B), and markedly decreased the expression of β-catenin (Figure 9C) and vimentin (Figure 9D). These results suggest that CP and NEM can modulate the EMT of HT-29 and LoVo cell lines. Furthermore, significant differences in EMT-related markers expression were observed between CRC treated cells.

## 3. Discussion

Propolis has attracted considerable attention from the medical and scientific community for its encouraging properties as an alternative therapy for cancer treatment [19,25,42]. In this study, brown Cuban propolis and its main component NEM were evaluated for their ability to affect migratory and metastatic capacities of colorectal cancer cells.

Malignant cancer cells develop an uncontrolled proliferation, which causes resistance to programmed cell death. Therefore, inhibition of cell proliferation and induction of apoptosis are anticancer mechanisms exploited by many chemotherapeutic drugs [43,44]. This study clearly showed that brown Cuban propolis and NEM displayed a pronounced inhibitory effect on the proliferation of HT-29 and LoVo cells. Furthermore, according to others reports [32,36,38], considering the NEM content of CP and the IC_50_ values obtained in this study, we further confirmed that the biological activity of CP is strictly dependent on its nemorosone content.

Their effect on HT-29 and LoVo viability was supported by the results of the clonogenic assay, which showed a significant decrease of the colony-forming capacity of CP- and NEM-treated cells compared to untreated cells, further confirming their antiproliferative effect. We have previously reported that CP and NEM have an inhibitory effect on LoVo cell viability [32], thus the results of the present study confirmed this finding and extended it to another CRC cell line. On the other hand, we observed differences in the susceptibility of these two CRC cell lines after 10 days of treatment with NEM, which was more active against LoVo than HT-29 when used at the lowest concentration. According to Zhu and collaborators [45], the expression of a mutant p53 (Arg273His) in HT-29 cells and not in LoVo cells, which express wild-type p53, could help the development of several cellular pathways involved in cell survival and chemoresistance. In this regard, the greater sensitivity of LoVo cells when compared with HT-29 cells after 10 days of treatment can probably be attributed to the expression of p53 mutants.

Cell cycle alteration is a critical step in the arrest of CRC progression [46]. In the present study, we observed that the antiproliferative effect of NEM and CP could be ascribable to the inhibition of cell cycle progression with an arrest in the G0/G1 phase and the induction of cell death by apoptosis. Both CP and NEM induced apoptosis in CRC cell lines in a time-dependent manner. In addition, the occurrence of apoptosis was associated with the upregulation of apoptosis-related genes *TP53* and *BAX*, the downregulation of *BCL2* and the activation of caspase-3. A previous study performed in our laboratory revealed that NEM and CP are able to induce mitochondrial dysfunction in LoVo cells [32]. The present findings were therefore in line with our previous results, thereby confirming that NEM and CP induced cell death via the mitochondrial apoptotic pathway in CRC. 

Migration and invasiveness of cancer cells are the main processes of tumor metastasis, which is the most frequent cause of colon cancer-associated death [7]. In order to evaluate the antimetastatic effect of NEM and CP on CRC cell lines, the wound healing and transwell migration/invasion assays were performed. Our results indicated that NEM and CP suppressed migration and invasion of both CRC cell lines. Interestingly, the LoVo cell line showed greater migratory and invasive capacity with respect to HT-29 cell line. This is probably due to a different genetic background of the two CRC cell lines [40,41], since LoVo cells derived from a metastatic site, while HT-29 cells derived from colon epithelial tissue [47,48]. Thus, it is reasonable to hypothesize that the difference between the effect of NEM and CP on cell migration and invasion capacity of the two CRC lines could be due to their peculiar genomic pattern, giving strong migratory properties to LoVo cells.

It is reported that the activity of proteolytic enzymes, such as the metalloproteinase MMP9, plays an important role in metastasis, since it is associated with the degradation of the extracellular matrix (ECM), which is involved in invasion/metastatic process [49]. We demonstrated that MMP9 activity was significantly reduced by the treatment with NEM and CP. Since it is well known that the expression of MMP-9 is correlated with angiogenesis and metastasis of CRC [50], we hypothesized that NEM and CP could affect not only the MMP9 expression in colon cancer cells, but also the expression of EMT-related molecules such as e-cadherin, β-catenin and vimentin, all involved in the migration, invasion and angiogenesis of CRC cells. In particular, e-cadherin is an important regulator of EMT process, and its expression decreases when epithelial cells lose cellular adhesiveness and acquire an aggressive and invasive phenotype [51]. β-catenin forms a junctional complex with e-cadherin; therefore, a decrease in e-cadherin expression results in an increase in β-catenin levels, which activate different proliferation pathways and promote cell migration [52]. Moreover, the expression of vimentin, a microtubular component, is increased when the cell–cell adhesion is lost, and the cells acquire a migratory and invasive behavior [53]. Consequently, interfering with the expression or function of these EMT markers could contribute to suppress tumor aggressiveness [54]. In this study, we observed that HT-29 and LoVo cell lines exhibited an increase in E-cadherin protein levels and a decrease of β-catenin and vimentin expression following treatment with NEM and CP.

Although different reports have already demonstrated that propolis has an antitumoral activity both in vitro and in vivo by inhibiting tumor proliferation, inducing cell death [25], and decreasing migration and invasion of tumor cells [55], this study provides for the first time evidences of the antimetastatic potential of brown Cuban propolis and in particular of NEM, and correlates this activity to the modulation of markers associated with the epithelial–mesenchymal transition. 

## 4. Materials and Methods

### 4.1. Chemicals and Antibodies

Dulbecco’s modified eagle medium (DMEM), Ham’s F12 medium and fetal bovine serum (FBS) were purchased from BioWhittaker® Reagents, Lonza (Blacklay, United Kingdom). Dulbecco’s phosphate-buffered saline (DPBS), L-glutamine and Trypsin-EDTA were purchased from EuroClone (Milano, Italy). Dimethylsulfoxide (DMSO), Streptomycin-penicillin, 3-(4, 5-dimethylthiazol-2-yl)-2, 5- diphenyltetrazolium bromide (MTT), crystal violet, gelatin, paraformaldehyde (PFA), Coomassie blue (R250) and ethanol were purchased from Sigma Aldrich (St. Luis, MO, USA). Annexin V-FITC/PI kit and Hoechst 33,342 was supplied by Invitrogen (San Diego, CA, USA). Matrigel matrix was supplied by Corning (Corning, NY, USA). Caspase-Glo 3/7 kit was provided from Promega (Milan, Italy). Nemorosone (1-benzoyl-4-hidroxy-8,8-dimethyl-3,5,7-tris (3-methyl-2-butenyl)-bicyclo-[3.3.1]-non-3-ene-2,9-dione) was provided by the Pharmacy and Food Institute (Havana, Cuba) and Cuban propolis (CP) extract classified as brown propolis (CBP17) was obtained from Estación Experimental Apícola (Havana, Cuba) [35]. Antibodies: anti-E-cadherin (sc-56527) and antivimentin (sc-6260) were purchased from Santa Cruz Biotechnology (Santa Cruz, CA, USA). Anti-β-catenin (bs-1165R-TR) was provided by Bioss Antibodies (Boston, MA, USA). The secondary antibodies Alexa-Fluor anti-mouse 568-conjugated and Alexa-Fluor anti-rabbit 488-conjugated were purchased from Abcam (Cambridge, UK).

### 4.2. Sample Preparation

A stock solution of CP was prepared by dissolving 10 mg of dry extract into 250 μL of PBS, whereas NEM stock solution was prepared by dissolving 1 mg into 1 mL of PBS containing 25% DMSO. The cell treatment was freshly prepared by diluting stock solutions in a complete medium. DMSO concentration in cell treatments was always below 0.025%. CP NEM content was 33.8 μg/mg [32,56]. 

### 4.3. Cell Culture

Human colorectal adenocarcinoma HT-29 (ATCC-HTB-38™) and LoVo (ATCC-CCL-229™) cells were maintained in DMEM and Ham’s F12 media, respectively. The culture media were supplemented with 1% L-glutamine, 1% streptomycin/penicillin and 10% FBS. The cell lines were maintained at 37 °C and 5% CO_2_.

### 4.4. Cell Viability Assay

Viability of HT-29 and LoVo cells was evaluated by means of the MTT assay. Cells were seeded into 96-well plates (10^5^ cell/mL). The effect of NEM (5, 10, 25 and 50 µM) and CP (6.25, 12.5, 25, 50 and 100 µg/mL) on cell viability was determined after 24, 48 and 72 h of exposure according to a previously published method [57]. The 50% inhibitory concentration (IC_50_) was calculated by means of the software GraphPad Prism ver. 8.0 by the appropriate nonlinear regression curve fit. 

### 4.5. Colony Formation Assay

The effect of NEM (5, 10, 25 and 50 µM) and CP (6.25, 12.5, 25, 50 and 100 µg/mL) on the colony formation capacity of HT-29 and LoVo cells was evaluated by the clonogenic assay [58]. Briefly, cells were seeded into 12-well plates (2 × 10^2^ cells/mL) overnight. After 10 days of exposure to NEM or CP, the cells were fixated with 4% paraformaldehyde (PFA) and stained with 1% crystal violet. The colonies (N cells ≥ 50) were counted using Image J software. Surviving fraction (SF) was determined using the formula: SF = (N° of colonies)/(N° of inoculated cells) ×100.

### 4.6. Cell Cycle Assay

Cell cycle analysis was performed by means of a flow cytometer (Epics XL-Beckmann Coulter and CXP software (San Diego, CA, USA). HT-29 and LoVo cells were seeded into 12-well plates (10 × 10^4^ cells/mL) and treated with NEM or CP (IC_50_) for 24 h and 48 h. After fixation with 70% ethanol for 15 min at 4 °C, cells were centrifuged for 5 min at 1800 rpm and stained with propidium iodide (PI, 300 μL) for 30 min.

### 4.7. Annexin V-FITC/PI Assay

HT-29 and LoVo cells were seeded into 12-well plates (10 × 10^4^ cells/mL) and treated with NEM or CP (IC_50_) for 24 and 48 h. The Annexin V-FITC/PI staining was performed according to manufacturer’s instructions and flow cytometry was used to determine percentage of apoptotic cells.

### 4.8. RNA Extraction and Quantitative RT-PCR (qRT-PCR)

Cells were seeded into 12-well plates (10 × 10^5^ cells/mL) and treated with NEM or CP (IC_50_) for 24 h. Total RNA was isolated using the kit Isolate II RNA (Bioline, London, UK), according to the manufacturers’ instruction, and a DNAse treatment was performed to avoid DNA contamination. qRT-PCR was performed as previously described [59], using One Step SYBR Prime Script RT-PCR Kit (Takara, Mountain View, CA, United States) and primers reported in Table 2. mRNA relative expressions were calculated using the 2^−ΔΔCt^ method [60] and GAPDH as the housekeeping gene. 

### 4.9. Caspase-Glo 3/7 Assay

HT-29 and LoVo cells were seeded into 12-well plates (10 × 10^5^ cells/mL) and treated with NEM or CP (IC_50_) for 24 and 48 h. Caspase-Glo 3/7 assay was performed according to manufacturer’s instructions. Caspase activity was normalized with the number of live cells determined by trypan blue staining.

### 4.10. Wounding Healing Assay

Cells were plated into 24-well plates (10 × 10^4^ cells/mL) using a serum-free medium. After 24 h, pipette tips were used to scratch the cell monolayer. The wound was washed to remove non-adherent cells and treated with NEM or CP (IC_50_) for 24 h. Cell migration into the scratched area was observed using the microscope Nikon T-s (10× magnification) and quantified using Image J software. The percentage of wound closure was determined as:

%Wound closure = (Area of wound (0h) - Area of wound (24 h))/Area of wound (0 h) × 100

### 4.11. Cell Migration and Invasion Assays

Migration and invasion assays were performed using 24-well Transwell plates with 8 μm membrane insert (Corning Inc., Lowell, MA, USA). For invasion assay, the membrane inserts were pre-coated with Matrigel. HT-29 and LoVo cells (10 × 10^4^ cells/mL) were placed in serum-free medium into the upper well and complete medium with or without NEM or CP (IC_50_) was added into the lower chamber. After 24 h of incubation, the cells on the upper surface of the insert were removed using a cotton swab. The migrated/invaded cells were fixed with 4% PFA and stained with crystal violet (0.2%). The migrated/invaded cells were quantified using a Nikon T-s microscope (10× magnification) and Image J software.

### 4.12. Metalloproteinase Activity

Metalloproteinase activity was determined by zymography [61]. Cells were seeded into 12-well plates (10 × 10^5^ cells/mL) and treated with NEM or CP (IC_50_) for 24 h. After treatment, total lysates were prepared as previously described. Of total lysate 15 µg was run into a polyacrylamide gel (8%) containing 1% gelatin. The gel was washed with 2.5% Triton X-100 and incubated overnight with developing buffer [62]. Coomassie blue (R250) was used to stain the gel. Then, the clear bands corresponding to MMPs were revealed using a destaining solution (5% acetic acid and 10% methanol) and quantified by means of Image J software.

### 4.13. Immunocytochemistry (ICC)

Cells were seeded into 24-well plates (10 × 10^4^ cells/mL). After 24 h-treatment with NEM or CP (IC_50_), cells were washed with PBS and fixed with 4% PFA for 30 min [59,63]. Then, 10% FBS was added for 20 min to block cross reaction with nonspecific ligands. Cells were then washed with PBS and incubated overnight with primary antibodies against E-cadherin, cell membrane β-catenin and Vimentin (dilution 1:200). After PBS wash, cells were incubated for 1 h with antimouse Alexa Fluor 568-conjugated (dilution 1:200) or antirabbit Alexa Fluor 488-conjugated (dilution 1:200) secondary antibodies. Hoechst 33,342 was used as nuclear dye. The images of the immunostained cells were acquired by means of a confocal microscope Zeiss LSM 800. ImageJ software was used to quantify the intensity of the fluorescent signal [64].

### 4.14. Statistical Analysis

All experiments were run in triplicate and the results were expressed as mean ± S.D. One-way analysis of variance (ANOVA) followed by the post hoc Tukey’s test for multiple comparisons was performed by means of the GraphPad Prism software (v:8, San Diego, CA, USA). *P* < 0.05 was considered statistically significant [65]. 

## 5. Conclusions

Our study demonstrated that brown Cuban propolis and its main component nemorosone inhibited cell viability and clonogenic capacity of HT-29 and LoVo cell lines, since they induced apoptosis-mediated G0/G1 phase cell cycle arrest, upregulated *TP53* and *BAX* gene expression and downregulated that of *BCL2*. In addition, we demonstrated that the apoptotic intrinsic pathway was involved. Furthermore, CP and NEM suppressed cell migration and invasion of CRC cells by inhibiting of EMT-related markers expression. In conclusion, we provided new insights into the anticancer mechanism of NEM, in particular about its antimetastatic effect, thereby confirming its promising pharmacological features for CRC therapy. Further in vivo studies are needed to assess the translational relevance of our findings. 

## Figures and Tables

**Table 1:** IC_50_ values of NEM and Cuban propolis (CP) samples.

	NEM (µM)		CP (µg/mL)	
	HT-29	LoVo	HT-29	LoVo
24 h	57.1 ± 3.7	64.3 ± 4.7	83.0 ± 8.2	73.8 ± 5.7
48 h	33.4 ± 2.8 ^**^	35.9 ± 9.1 ^&&^	39.9 ± 8.9 ^***^	43.7 ± 6.3 ^&^
72 h	25.7 ± 3.3 ^***/$^	22.8 ± 6.2 ^&&&/#^	20.2 ± 6. 8 ^****/$^	24.9 ± 10.7 ^&&&/#^

Data are expressed as mean ± S.D. HT-29 cells: ** *p* < 0.01; *** *p* < 0.001, **** *p* < 0.0001 vs. 24 h. ^$^ *p* < 0.05 vs. 48 h; LoVo cells: ^&^ *p* < 0.05, ^&&^ *p* < 0.01, ^&&&^ *p* < 0.001 vs. 24 h. ^#^ *p* < 0.05 vs. 48 h.

**Table 2:** Primer sequences.

Gene	Forward Sequence	Reverse Sequence
***GAPDH***	ACATCAAGAAGGTGGTGAAGCA	GTCAAAGGTGGAGGAGTGGGT
***TP53***	GAGACCTGTGGGAAGCG	CGGGGACAGCATCAAAT
***BAX***	CACTGAAGCGACTGATGTCCC	CCGCCACAAAGATGGTCAC
***BCL2***	TGTGTGTGGAGAGCGTCAA	CAGCCCAGACTCACATCACCA
